# Differential response of serum amyloid A to different therapies in early rheumatoid arthritis and its potential value as a disease activity biomarker

**DOI:** 10.1186/s13075-016-1009-y

**Published:** 2016-05-17

**Authors:** Yong Gil Hwang, Goundappa K. Balasubramani, Ilinca D. Metes, Marc C. Levesque, S. Louis Bridges, Larry W. Moreland

**Affiliations:** Department of Medicine, Division of Rheumatology and Clinical Immunology, University of Pittsburgh, 3500 Terrace Street, Pittsburgh, PA 15261 USA; Department of Epidemiology, School of Public Health, University of Pittsburgh, 130 DeSoto Street, 127 Parran Hall, Pittsburgh, PA 15261 USA; Department of Medicine, Division of Clinical Immunology and Rheumatology Birmingham, University of Alabama at Birmingham, Shelby Building, Room 178B, 1825 University Blvd., Birmingham, AL 35294-2182 USA; AbbVie Inc, 100 Research Dr, Worcester, MA 01605 USA

**Keywords:** Serum amyloid A, C-reactive protein, Early rheumatoid arthritis, Etanercept

## Abstract

**Background:**

The aim was to compare the effect of etanercept (ETN) and conventional synthetic disease-modifying anti-rheumatic drug (DMARD) therapy on serum amyloid A (SAA) levels and to determine whether SAA reflects rheumatoid arthritis (RA) disease activity better than C-reactive protein (CRP).

**Methods:**

We measured SAA and CRP at baseline, 24, 48, and 102 week follow-up visits in 594 patients participating in the Treatment of early RA (TEAR) study. We used Spearman correlation coefficients (rho) to evaluate the relationship between SAA and CRP and mixed effects models to determine whether ETN and methotrexate (MTX) treatment compared to triple DMARD therapy differentially lowered SAA. Akaike information criteria (AIC) were used to determine model fits.

**Results:**

SAA levels were only moderately correlated with CRP levels (rho = 0.58, *p* < 0.0001). There were significant differences in SAA by both visit (*p* = 0.0197) and treatment arm (*p* = 0.0130). RA patients treated with ETN plus MTX had a larger reduction in SAA than patients treated with traditional DMARD therapy. Similar results were found for serum CRP by visit (*p* = 0.0254) and by treatment (*p* < 0.0001), with a more pronounced difference than for SAA. Across all patients and time points, models of the disease activity score of 28 joints (DAS28)-erythrocyte sedimentation rate (ESR) using SAA levels were better than models using CRP; the ΔAIC between the SAA and CRP models was 305.

**Conclusions:**

SAA may be a better biomarker of RA disease activity than CRP, especially during treatment with tumor necrosis factor (TNF) antagonists. This warrants additional studies in other cohorts of patients on treatment for RA.

**Trial registration:**

(ClinicalTrials.gov identifier: NCT00259610, Date of registration: 28 November 2005)

## Background

Serum levels of systemic acute-phase reactants (APR) such as C-reactive protein (CRP), and serum amyloid A (SAA) are increased during active synovitis, and APR are known to be significantly associated with joint damage and disability on long-term follow up [1–4]. Therefore, measurement of serum APR is commonly used as an indicator of disease severity, progression and prognosis in rheumatoid arthritis (RA).

The widely used disease activity score of 28 joints (DAS28) is based on a count of 28 swollen and tender joints, a measure of general health or global disease and an inflammatory marker. The DAS28 based on erythrocyte sedimentation rate (DAS28-ESR) has been extensively validated for use in clinical trials in combination with the European League Against Rheumatism (EULAR) response criteria [5, 6]. However, CRP and SAA have many advantages over ESR, which is heavily influenced by age and sex and has a slow response to change [7, 8]. Therefore, an alternative formulation of the DAS28 based on CRP (DAS28-CRP) was developed and validated against radiographic progression and physical function [9]. However, there is a considerable variation in CRP levels in patients with RA, and a substantial proportion of patients with RA have a clinically insignificant range of values [10–13]. It has been recognized that DAS28-CRP tends to yield lower values of disease activity than the DAS28-ESR, resulting in substantial classification differences [9, 14, 15]. In addition, as DAS28 based on ESR or CRP integrates various aspects of the disease into a single numerical value, there can be a great discrepancy between patient and provider assessments of disease activity. RA disease activity cannot be measured accurately by a single appropriate disease activity measure and the ideal targets of therapy remain elusive.

In contrast to ESR or CRP, less is known about the actions of SAA protein and the clinical usefulness of SAA in RA. SAA is an acute-phase protein linked to the pathogenesis of various diseases, such as atherosclerosis, diabetes, Alzheimer's disease, and RA [16]. Clinical studies have shown that SAA could be a better predictor of clinical outcomes than CRP in non-ST elevated acute coronary syndromes or end-stage renal disease and a better early predictor of severity in acute pancreatitis [17–19]. SAA was originally of interest to investigators studying amyloidosis, because chronic elevation of SAA in patients with RA could lead to amyloid deposition, resulting in systemic amyloidosis with major organ damage [20]. Growing evidence suggests that acute-phase SAA is sensitive to change, reaches much higher levels than CRP, declines rapidly, and may therefore accurately reflect disease activity [17, 19, 21]. In relation to diagnosis, disease activity, and assessment of treatment response in RA, SAA has been shown correlate well with disease activity, and decreases in serum SAA can be useful in predicting clinical response [22–25]. However, there is a lack of clinical studies in large groups of patients with RA to assess the value of SAA in monitoring disease activity and predicting treatment response with various treatments, such as traditional disease-modifying anti-rheumatic drug (DMARD) combination therapy or biologic therapy. It remains unclear how well SAA and CRP correlate with disease activity during treatment and whether SAA is better or the same as a biomarker of disease activity during therapy.

The aim of this study was to investigate whether patients with RA treated with the TNF antagonist etanercept (ETN) have greater reductions in SAA than patients with RA treated with traditional oral DMARDs, when both treatments result in nearly equivalent reductions in disease activity [26]. We also explored whether SAA models RA disease activity better than CRP, especially during treatment with a TNF antagonist. Therefore, we compared changes in SAA levels with CRP levels in relation to disease activity measured by DAS28-ESR and treatment modalities using data from the Treatment of early RA (TEAR) study.

## Methods

### Patients

All study serum samples were obtained from subjects enrolled in the TEAR study, a randomized, double-blinded comparative effectiveness trial [26]. The TEAR study enrolled subjects older than 18 years who was diagnosed with RA according to the 1987 American College of Rheumatology (ACR) criteria [27, 28]. They also had active disease (at least 4 swollen joints and 4 tender joints, using a 28-joint count); positivity for rheumatoid factor (RF) or anti-cyclic citrullinated peptide (anti-CCP) antibodies, or if seronegative, the presence of ≥2 erosions on radiographs of the hands/wrists/feet [26]. The main objective of the TEAR trial was to determine whether immediate and aggressive combination drug therapy was more effective in controlling early RA disease activity and underlying symptoms than methotrexate (MTX) monotherapy and subsequent step-up treatment.

The TEAR trial was also performed to evaluate the comparative effectiveness of two combination drug therapies. It was designed so that patients with early RA (defined as patients with disease duration <3 years and <2 months of prior DMARD therapy), active (at least 4 swollen joints and 4 tender joints using a 28-joint count) were randomized to receive MTX monotherapy, combination ETN and MTX therapy (ETN/MTX), or combination MTX/hydroxychloroquine (HCQ)/sulfasalazine (SSZ) triple oral therapy. Subjects assigned to the MTX monotherapy arm with sustained RA disease activity (designated as having a DAS28-ESR >3.2 after 6 months of treatment) were randomly assigned to step up therapy and received either combination ETN/MTX or triple oral therapy. All centers participating in the TEAR trial received local Institutional Review Board or Western Institutional Review Board approval and informed consent was obtained from all study participants [26]. The members of the TEAR study group are shown in Acknowledgements.

### Serum SAA and CRP measurements

The TEAR serum samples and clinical data including demographic information, body mass index (BMI) measured in kg/m^2^, rheumatoid factor (RF) positivity, prior DMARD use, glucocorticoid use, and formal counts of swollen and tender joints in 28 joints used in this study were obtained when treatment was initiated and at follow up visits at weeks 24, 48, and 102. CRP at baseline was available in 594 subjects and SAA at baseline were available in 559 subjects. There were 546 subjects with both CRP and SAA measurements at baseline. CRP (mg/L) was measured in plasma samples at the Clinical and Epidemiological Research Laboratory at the Children’s Hospital in Boston using a high-sensitivity immunoturbidimetric assay on a Hitachi 917 autoanalyzer (Roche Diagnostics, Indianapolis, IN, USA), with reagents and calibrators from Denka Seiken (Tokyo, Japan) [29] as previously reported [30]. High CRP was defined as >3 mg/L [29]. We measured plasma rather than serum CRP because of the availability of plasma specimens in our study population and because the measurement of CRP in plasma and serum are comparable [31]. SAA (mg/L) was measured in serum samples at Clinical Biochemistry Research at the University of Vermont using the Seimens BNII nephelometer (Siemens Healthcare Diagnostics, Deerfield, IL, USA). The lowest detection limit for SAA was 0.97 mg/L, determined by the lower limit of the reference curve [32].

### Statistical analysis

Summary statistics are presented as means and standard deviations for continuous variables, and percentages for discrete variables. Analysis of variance (ANOVA) one-way classification was used to compare continuous baseline clinical and demographic measures in the treatment groups. The chi-square test was used to compare discrete characteristics in the treatment groups.

Spearman correlation coefficients (rho) were first calculated to determine the overall correlation between SAA and CRP. To best model the relationship of SAA and CRP, the Spearman coefficients were measured first looking at each individual study time point while including all study participants regardless of treatment, and then additionally determining the relationship at each study time point while grouping the participants by treatment arm.

To better understand the possible treatment effects on SAA levels over time, a repeated-measures, mixed-effects model was used to determine whether ETN/MTX combination therapy differentially lowered SAA from the follow up at week 24 to the follow up at week102. SAA level was the outcome variable of the mixed model with the fixed effects considered being treatment (ETN/MTX, triple oral therapy, and step-up MTX monotherapy), the study time point (weeks 24, 48, and 102), baseline ranked SAA value, DAS28-ESR to control for disease activity, and the differential effect of treatment and time that included each possible treatment arm and each possible study time point. To address the possible treatment effects on CRP over time, another repeated-measures, mixed-effects model was used to determine whether ETN/MTX combination therapy differentially lowered CRP from follow up at week 24 to follow up at week 102. CRP level was the outcome variable for the second mixed model, with the fixed effects considered being treatment (ETN/MTX, triple oral therapy, and step-up MTX monotherapy), the study time point (weeks 24, 48, and 102), baseline rank CRP value, DAS28-ESR to control for disease activity, and the differential effect of treatment and time interactions that included each possible treatment arm and each possible study time point.

Last, to address the second objective of the study and to better understand whether SAA levels might model RA disease activity (as measured by the DAS28-ESR) better than the more traditionally used CRP, additional repeated-measures, mixed-effects models were conducted to determine the fit between (1) SAA and the DAS28-ESR, and (2) CRP and the DAS28-ESR for patients being treated with ETN/MTX combination therapy versus patients being treated with oral DMARDs. DAS28-ESR score was the outcome variable for these additional mixed models, with the fixed effects including treatment and baseline rank SAA and baseline rank CRP values, respectively. Two commonly used model selection criteria, Akaike information criteria (AIC) and Bayesian information criteria (BIC) were used to determine the model fit. The best model in the group compared is the one that minimizes these scores, in both cases.

## Results

A total of 755 subjects with RA enrolled in the TEAR study were analyzed when treatment was initiated and at follow-up visits at 24, 48 and 102 weeks. Subjects were randomized to one of four treatment arms: immediate treatment with MTX plus ETN (*n* = 244), immediate oral triple therapy (MTX plus SSZ plus HCQ) (*n* = 132), or step up from MTX monotherapy (*n* = 379) to one of the combination therapies (either MTX plus ETN (*n* = 205) or MTX plus SSZ plus HCQ (*n* = 93)) at week 24 (beginning of the step-up period) if the DAS28-ESR was ≥3.2. Overall results from the TEAR trial showed that after 2 years of treatment there were equal reductions in RA disease activity as measured by the DAS28-ESR in all treatment arms of the study, although subjects in the two immediate combination treatment groups demonstrated a greater reduction in the DAS28-ESR compared with those in the two step-up groups at week 24. This study was performed in subjects who consented to participation in the biorepository (*n* = 594), which was similar to the overall group of TEAR participants (data not shown).

**Fig. 1:**
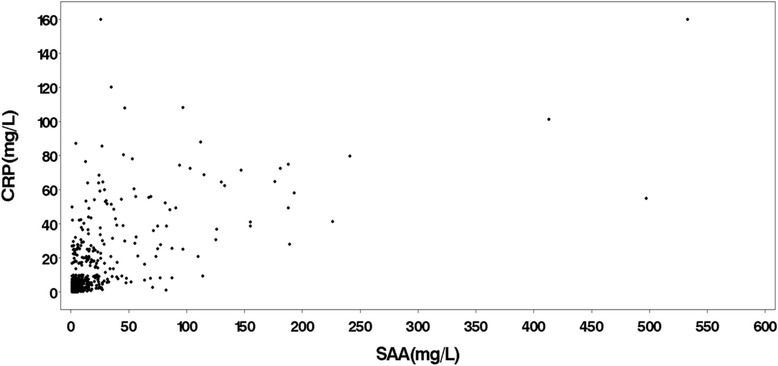
Serum amyloid A (*SAA*) (mg/L) and C-reactive protein (*CRP*) (mg/L) at the baseline study visit

There were no baseline differences in age, SAA, CRP, RF status, or disease duration between the different treatment arms of the TEAR trial (Table 1). Of the patients included, 72 % were female, and the mean disease duration was 3.6 years (SD 6.5). Subjects had moderate to severe disease activity (DAS28-ESR 5.8 ± 1.1) at the time of enrollment. At the baseline visit, SAA and CRP were 14.1 (±23.1) and 22.4 (±72.8), respectively. At the initial visit, ESR was better correlated with CRP (rho = 0.54) than with SAA (rho = 0.29), and SAA was moderately correlated with CRP (rho = 0.58) at the initial visit. At follow-up visits, the distributions of circulating levels of CRP and SAA were markedly skewed to the right and the majority of subjects (*n* = 350 (65.1 %)) had low values of both (CRP <10 mg/L, SAA <20 mg/L). There were 49 subjects (9.0 %) with high values of both (CRP >20 mg/L, SAA >40 mg/L); 50 subjects (9.2 %) had high CRP (>20 mg/L) and low SAA (<20 mg/L) but only 12 subjects (2.2 %) had high SAA with low CRP (Table 2 and Fig. 1). The correlation between SAA and CRP was similar regardless of treatment or study visit (rho range 0.35–0.62).

**Table 1:** Baseline demographic and clinical characteristics by treatment group

	Overall	Treatment group						*P* value
		Immediate combination therapy		Step up from MTX monotherapy				
		AE	AT	MTX (SE)	SE	MTX (ST)	ST	
Age, mean ± SD, years	49.3 ± 12.7	50.5 ± 13.4	49 ± 12.7	47.7 ± 11.9	48.9 ± 12.9	47.6 ± 12.8	48.7 ± 11.1	0.69
Female sex, *n* (% of total)	404 (72.3)	142 (73.6)	72 (75.8)	23 (67.6)	101 (69.7)	14 (58.3)	52 (76.5)	0.47
BMI, mean ± SD, kg/m^2^	30.1 ± 7.5	29.7 ± 7.2	30.4 ± 8.7	29.3 ± 5.4	30.9 ± 7.1	30 ± 5.9	29.7 ± 8.4	0.73
Disease duration, mean ± SD, years	3.5 ± 6.4	3.4 ± 6.2	3.9 ± 7.1	2.8 ± 5.8	3 ± 5.4	4.5 ± 7.3	4.6 ± 7.5	0.47
RF-positive, *n* (% of total)	495 (88.6)	168 (87.1)	88 (92.6)	31 (91.2)	130 (89.7)	20 (83.3)	58 (85.3)	0.59
DAS28-ESR, mean ± SD	5.8 ± 1.1	5.8 ± 1.1	5.7 ± 1.1	5.5 ± 1.0	5.9 ± 1.1	5.6 ± 0.9	6 ± 1.1	0.20
Baseline SAA, *n* (mean ± SD), mg/L	559 (22.4 ± 72.8)	193 (34.1 ± 116.6)	95 (17.4 ± 33.8)	34 (13 ± 14.4)	145 (16.1 ± 24.7)	24 (12.3 ± 38)	68 (17.9 ± 31.3)	0.16
Baseline CRP, *n* (mean ± SD), mg/L	594 (14.1 ± 23.1)	194 (16 ± 25.4)	104 (14.3 ± 26.1)	36 (9.7 ± 12.1)	159 (12.9 ± 20.5)	25 (8 ± 9)	76 (15.9 ± 24.8)	0.38

Among participants in the combination arm: *AE* immediate treatment with methotrexate (*MTX*) + etanercept (*ETN*), *AT* immediate oral triple disease-modifying anti-rheumatic drug (DMARD) therapy (MTX + sulfasalazine + hydroxychloroquine). Among participants in the MTX monotherapy arm: *MTX (SE)* MTX only without step-up to MTX + ETN at week 24 SE): step-up to MTX + ETN at week 24; *MTX (ST)* MTX only without step-up to triple DMARD therapy at week 24; *ST* step-up to triple DMARD therapy at week 24. BMI body mass index, *SAA* serum amyloid A, *RF* rheumatoid factor, *DAS28-ESR* disease activity score in 28 joints-erythrocyte sedimentation rate, *CRP* C-reactive protein

**Table 2:** Distribution of serum serum amyloid A (SAA) and C-reactive protein (CRP) at the baseline study visit

CRP (mg/L)		SAA(mg/L)			
		<20	20-40	>40	Total
<10	Frequency (n)	350	26	12	390
	Row (%)	89.7	7.2	3.1	100
	Column (%)	83.5	43.1	19.4	71.4
10-20	Frequency (n)	19	12	1	32
	Row (%)	59.4	37.5	3.1	100
	Column (%)	4.5	18.5	1.6	5.9
>20	Frequency (n)	50	25	49	124
	Row (%)	40.3	20.2	39.5	100
	Column (%)	11.9	38.5	79.0	22.7
Total	Frequency (n)	419	65	62	546
	Row (%)	76.7	11.9	11.4	100
	Column (%)	100	100	100	100

**Fig. 2:**
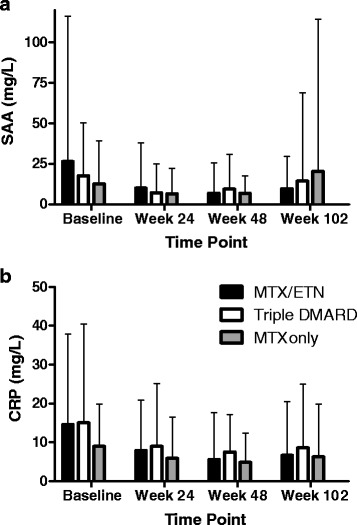
Mean serum amyloid A (*SAA*) (**a**) and C-reactive protein (*CRP*) (**b**) by treatment modality and study visit. *MTX* methotrexate, *ETN* etanercept, *DMARD* disease-modifying anti-rheumatic drug. *Error bar* denotes standard deviation

Overall, there were significant differences in SAA by both visit (*p* = 0.0197) and treatment arm (*p* = 0.0130). Patients with RA treated with ETN/MTX had a greater reduction in SAA than patients treated with oral DMARD combination therapy even after correcting for disease activity. SAA was lower by an average of 66 ranks following treatment with ETN/MTX compared to triple oral therapy, using the mixed-effects model. The results were similar for serum CRP both by visit (*p* = 0.0254) and by treatment arm (*p* < 0.0001) (Fig. 2). Serum CRP was also lower following treatment with ETN/MTX versus triple oral therapy. With an even more pronounced mean difference than SAA, serum CRP was lower by an average of 144 ranks following treatment with ETN/MTX compared to triple oral therapy (Fig. 2).

Across all patients and time points, models of the DAS28-ESR using SAA were better than models using CRP (Table 3); lower values of the AIC and BIC indicated a better model fit. In particular, AIC takes into account the goodness of fit and the number of parameters required to achieve this. The AIC is based on the likelihood function and provides the goodness of fit for the model. In Table 3, comparing the AIC values for the overall model ESR versus SAA (AIC = 6854) with ESR versus CRP (AIC = 7159.3) indicates that the former model provides the better fit (the ΔAIC between the models was 305). The model of DAS28-ESR using SAA was associated with an approximately sixfold better fit versus the CRP model for patients treated with ETN/MTX, and an approximately fivefold better fit versus the CRP model for patients treated with oral DMARDs (ΔAIC = 159 versus ΔAIC = 137, respectively).

**Table 3:** Determining model fit for rheumatoid arthritis disease activity (as measured by the DAS28-ESR) by serum SAA and CRP

	AIC fit statistic	ΔAIC	BIC fit statistic	Parameter estimate (SE)	*P* value
Overall					
DAS28-ESR vs. SAA	6854.0	305	6876.1	0.006(0.001)	<.0001
DAS28-ESR vs. CRP	7159.3		7181.5	0.038(0.002)	<.0001
By treatment – ETN					
DAS28-ESR vs. SAA	4068.2	159	4087.7	0.007(0.001)	<.0001
DAS28-ESR vs. CRP	4227.2		4246.8	0.041(0.003)	<.0001
By treatment – DMARD					
DAS28-ESR vs. SAA	2737.6	137	2762.2	0.006(0.002)	0.0007
DAS28-ESR vs. CRP	2875.0		2899.7	0.029(0.003)	<.0001

*AIC* Akaike information criterion, *BIC* Bayesian information criterion, *SE* standard error, *DAS28* disease activity score in 28 joints, *ESR* erythrocyte sedimentation rate, *SAA* serum amyloid A, *CRP* C-reactive protein, *ETN* etanercept, *DMARD* disease-modifying anti-rheumatic drug

## Discussion

For patients with RA in the TEAR trial, both CRP and SAA serum levels decreased over time with treatment, particularly in the MTX/ETN group. Patients with RA treated with ETN/MTX had a greater reduction in SAA than patients treated with oral DMARD therapy, even after correcting for disease activity. Our findings are consistent with previous studies that showed greater reduction in SAA with different combination therapies such as golimumab (golimumab + MTX vs MTX alone) or tofacitinb (tofacitinib + MTX vs tofacitinib monotherapy) [22, 33]. In an observational study of 50 consecutive patients with RA treated with long-term leflunomide, the reduction in SAA was transient, as it was no longer observed after 6 months, in spite of reduced disease activity [23]. In our study, the ETN/MTX treatment group had a sustained reduction in SAA by week 102, although the difference was not statistically significant.

Our study also compared SAA with CRP to study the usefulness of SAA for detecting systemic inflammation and for monitoring various treatments including anti-TNF treatment in early RA. The advantages of SAA as a biomarker of disease activity in early RA include the rapid production and exceptionally wide dynamic range of the SAA response [34, 35]. During acute inflammation, serum SAA may rise up to 1000-fold and the biologic half-life of SAA is significantly shorter than that of CRP [36, 37]. Connolly et al. showed that baseline SAA, but not ESR or CRP, correlates with the 28-joint swollen joint count and is independently associated with radiographic evidence of progression at 1 year [38]. In addition, SAA has a direct role in synovial inflammation and joint destruction [16, 38–42]. We demonstrated increased expression of SAA in patients with early active RA and observed moderate significant correlations between SAA and CRP regardless of treatment or study visit. In addition, SAA might model RA disease activity (as measured by the DAS28-ESR) better than CRP [34]. Therefore, elevated serum SAA can be a useful marker of disease activity and may be a more accurate indicator of clinical outcome in early RA [38].

Interestingly, the differential effect on SAA of ETN/MTX compared to triple oral DMARDs was less pronounced than the differential effects on CRP of ETN/MTX compared to triple oral DMARDs. The acute phase reaction is an integrated response involving a number of cytokines, hormones, steroids, and prostaglandins. SAA and CRP are synthesized under the influence of different cytokine combinations, and their function and contribution to the acute phase response may be very different [43, 44]. CRP is predominantly stimulated by interleukin-6, while SAA responds preferentially to interleukin-1 [39, 45]. Although CRP has been used widely to monitor disease activity in RA, TNF antagonist therapy (and likely therapy with other biologic agents) can reduce CRP, even without an associated reduction in RA disease activity. Our results suggest that SAA might model RA disease activity better and may be less affected by specific treatments with different mechanisms of action.

Our study has many limitations. Subjects in our study were limited to those with early active RA, which may limit the generalizability of our findings, especially in patients with long disease duration. There is no gold standard to measure RA disease activity objectively. Therefore, using DAS28-ESR as a gold standard to compare biomarkers has its own limitation. ESR is influenced by other factors not related to RA disease activity. The DAS28-ESR and DAS28-CRP definitions differ substantially in classifying patients with RA, with the ESR definition resulting in a higher proportion of patients with high DAS-28 especially among women [46]. In addition, the role of SAA in the pathogenesis of RA was based on a number of studies with recombinant SAA (rSAA). Although rSAA is a potent pro-inflammatory mediator, the present findings show that this activity is not shared by endogenous SAA [47, 48]. SAA is known to be less influenced by age and gender, but is determined by many other conditions such as metabolic syndrome, diabetes, dietary intake, etc., and the regulatory mechanism of SAA is not well-understood [49–52]. In our study SAA levels varied widely across patients and time points. Although there were statistically significant differences in SAA by both visit and treatment arm, differences in the ranks of SAA identified by nonparametric statistical tests may not be useful in daily practice, given the wide variation and right-skewed distribution. There were marked overlaps of the distributions of absolute values, which raises concern about the practical usefulness of SAA as a single biomarker for disease activity measurement (Fig. 2).

## Conclusions

With all limitations considered, the lack of strong correlation between SAA and CRP levels, less pronounced differential effects using biologic DMARDs and traditional DMARDs, coupled with their superior modeling of RA disease activity, suggests that SAA may be a better biomarker for RA disease activity than CRP, especially during treatment with TNF antagonists.

## Abbreviations

AIC: Akaike information criteria
ACR: American College of Rheumatology
ANOVA: analysis of variance
APR: acute phase reactant
BIC: Bayesian information criterion
BMI: body mass index
CRP: C-reactive protein
DAS28: disease activity score in 28 joints
DMARD: disease-modifying anti-rheumatic drug
ETN: etanercept
ESR: erythrocyte sedimentation rate
EULAR: European League Against Rheumatism
HCQ: hydroxychloroquine
MTX: methotrexate
RA: rheumatoid arthritis
RF: rheumatoid factor
SAA: serum amyloid A
SSZ: sulfasalazine
TEAR: Treatment of early RA study
TNF: tumor necrosis factor
